# Measuring Sleep at Home in Adolescents With Autism Using EEG Headbands: Protocol for a Remote Study

**DOI:** 10.2196/105941

**Published:** 2026-10-30

**Authors:** Paige Hickey Townsend, Lindsey Jones, Anabella Tolosa, Nicole Massa, Nicole A Simon, Dimitrios Mylonas, Ann M Neumeyer, Dara S Manoach

**Affiliations:** 1 Department of Psychiatry, Massachusetts General Hospital, Harvard Medical School, Boston, MA, United States; 2 Lurie Center for Autism, Massachusetts General Hospital, Lexington, MA, United States; 3 Department of Psychiatry, Massachusetts General Hospital, Boston, MA, United States; 4 Athinoula A. Martinos Center for Biomedical Imaging, Massachusetts General Hospital, 149 13th Street, Room 2618, Charlestown, MA, 02129, United States, 1 (617) 724-6148; 5 Department of Neurology, Harvard Medical School, Harvard University, Boston, MA, United States

**Keywords:** autism, sleep, electroencephalography, EEG, wearable device, desensitization

## Abstract

**Background:**

Sleep disturbances are prevalent in autism spectrum disorders (ASDs) and worsen daytime functioning, yet their neural bases remain poorly understood. This may reflect the challenges of laboratory-based polysomnography for individuals with ASD, including sensory sensitivities and difficulty adapting to changes in routine. The advent of wearable electroencephalography (EEG) devices allows for remote data collection in the home and facilitates large-scale sleep studies.

**Objective:**

The aim of this study is to test the hypothesis that thalamocortical circuit dysfunction contributes to poor sleep in ASD by characterizing sleep oscillations that rely on this circuitry. To achieve this goal, we monitored sleep in the home using wearable EEG headbands in adolescents with ASD, including those who are profoundly affected, and their typically developing peers. When necessary, we used a desensitization protocol to gradually acclimate participants to wearing the headband overnight to promote the inclusion of individuals with sensory sensitivities and high support needs.

**Methods:**

Participants and their families completed assessments and questionnaires and received remote training to use a wearable EEG headband. Those needing time to acclimate followed a personalized 2-week desensitization protocol with a practice headband. Sleep was monitored at home for 3 consecutive nights, the first of which was for adaptation to the headband. Sleep data quality was checked daily, and remote technical support was provided as needed. Using sleep rhythms as physiological biomarkers of thalamocortical circuit function, we will test the hypothesis that abnormal circuit function in ASD is related to worse sleep quality.

**Results:**

This study was funded by the Simons Foundation Autism Research Initiative (SFARI) and approved by the Mass General Brigham Institutional Review Board in November 2021. Data were collected between July 2023 and November 2025. A total of 146 participants (ASD: n=77; typically developing [TD]: n=65; profound autism: n=4) completed the study and produced at least 1 night of usable sleep data. Data analysis is underway, and the results will be submitted for publication in November 2026.

**Conclusions:**

At-home sleep monitoring enables large samples that are needed to characterize the neural underpinnings of sleep disturbances across the autism spectrum. The personalized desensitization protocol promotes inclusion by accommodating individuals often excluded from research due to sensory or behavioral challenges. These methods are adaptable for other conditions and research goals beyond ASD, such as longitudinal studies across development or characterizing sleep in other neurodevelopmental disorders. Desensitization protocols may also increase the tolerability of diagnostic laboratory-based sleep studies and other medical procedures, improving both patient experience and quality of care.

## Introduction

### Background

Autism spectrum disorder (ASD) is a neurodevelopmental disorder characterized by challenges with social interaction and communication and the presence of restricted interests, repetitive behaviors, or atypical sensory processing (ie, hyper- or hyposensitivities) [1]. Sleep disturbances afflict up to 80% of children with ASD [2] and exacerbate core symptoms [3,4], maladaptive behaviors [5,6], and learning and attention deficits [7]. Despite the links between poor sleep and worse daytime functioning, the neural mechanisms underlying sleep disturbances in ASD remain largely unknown. This reflects, in part, the difficulty of laboratory-based polysomnography (PSG) studies, which are costly and burdensome to both participants and staff. This paper describes the methodology of a study of sleep neurophysiology in adolescents with ASD that addresses these challenges by collecting sleep data in the home using wearable electroencephalography (EEG) headbands and implementing a desensitization protocol to acclimate participants to wearing the headband overnight. These methods are scalable, cost-effective, and facilitate larger, more inclusive sleep studies, including clinical trials. They hold the promise of illuminating the neural bases of sleep disturbances—a critical step toward the development of effective intervention.

### Prior Work

A growing body of work demonstrates that individuals with ASD exhibit abnormal brain rhythms during sleep, most notably a reduction in sleep spindles—a defining oscillation of nonrapid eye movement (NREM) sleep. Spindles are generated in the thalamus and are propagated to the cortex via thalamocortical circuitry, where they are often synchronized with cortically generated slow oscillations (SOs). The reduction in spindle density (number of spindles/min; [8-10]) in ASD suggests a dysregulation of thalamocortical interactions [11]. Given the thalamus’ role in gating the relay of sensory information from the senses to the cortex, abnormal thalamocortical interactions could contribute to both sleep disturbances and waking manifestations of ASD. Sleep spindles are theorized to protect the sleeper from being awakened by external noise [12]. Findings that brain responses to noise are dampened during spindles [13,14] and that people with fewer spindles are more likely to be awakened by noise [12] raise the possibility that the spindle deficit in ASD contributes to worse sleep quality. While awake, impaired sensory gating may contribute to sensory overload and sensitivities. Modulating thalamocortical interactions pharmacologically or via noninvasive brain stimulation has the potential to increase spindles and improve sleep quality and daytime functioning.

Sleep oscillations are traditionally recorded with laboratory-based PSG, which includes scalp EEG, electrooculography, and electromyography recordings. However, the laboratory setting and PSG equipment can be problematic for individuals with ASD. Children with ASD are 3 times more likely to have difficulty tolerating PSG [15] and take longer to acclimate to the equipment than their typically developing (TD) peers [15,16]. Disruption to nightly routines and the unfamiliar setting may trigger anxiety and discomfort [17]. People with ASD may also be particularly sensitive to “first night effects” to sleeping in a new environment, which include worse sleep quality and consequently reduced functioning the next day. One or more adaptation nights may be required to acclimate [18]. Overnight stays in a lab interfere with routines and can be logistically impractical for many families. Sleep studies are labor intensive for researchers, and the cost of equipment, personnel, and space can be prohibitive. Research teams may also lack experience working with individuals with behavior and communication impairments, which can make it difficult to anticipate and mitigate problems that arise.

For the approximately 26.7% of people with ASD who are profoundly affected [19] (ie, intellectual disability, minimal or no use of spoken language, and/or high daily support needs), sleep studies are even more challenging [20]. Their difficulty understanding and complying with instructions, and maladaptive (eg, aggression, self-injury, or meltdowns) or sensory behaviors (eg, mouthing or chewing), often lead to exclusion from research [21]. The proportion of studies including profound autism has decreased significantly since the 1990s across a range of disciplines [22]. Despite their significant sleep challenges [23,24], there are few EEG sleep studies in profound autism [8]. Their exclusion limits our understanding of the heterogeneity of sleep abnormalities and contributes to small sample sizes that do not represent the entire autism spectrum.

Wearable EEG headbands used at home offer a practical alternative to laboratory-based PSG, potentially broadening participation and inclusion in sleep studies of ASD. Commercially available sleep EEG headbands measure sleep quality and physiology and have been validated against PSG for sleep staging [25]. They are also relatively inexpensive, user-friendly, and more comfortable than PSG. Although an adaptation night is still advisable to acquire representative sleep data, home monitoring alleviates the anxiety associated with laboratory sleep studies and interferes less with nightly routines. For participants with sensory sensitivities, a structured desensitization protocol can increase the likelihood of successful study completion [16]. This involves gradually introducing the equipment over time in a series of incremental steps. This strategy has been useful for facilitating overnight PSG recordings [16,26]. Coupling EEG headbands with a desensitization protocol enables the inclusion of larger, more representative samples, which is necessary to understand sleep disturbances across the autism spectrum and to inform treatment development.

### Goal of This Study

The goal of this study is to characterize sleep oscillations in adolescents with ASD, including those who are profoundly affected. To achieve this goal, we monitored sleep in the home using a wearable EEG headband and, when necessary, used a desensitization protocol to gradually acclimate participants to wearing it overnight. We hypothesize that dysfunctional thalamocortical circuitry and the consequent spindle deficit underlie sleep disturbances in ASD. Using sleep spindles as physiological biomarkers of circuit function, we will test the predictions that this circuitry will be atypical in ASD (reflected as reduced spindle density) and correlate with worse sleep quality (ie, more wake after sleep onset [WASO] and a higher sleep fragmentation index).

## Methods

### Study Design

#### Overview

Participants (ages 12-19 years) or their caregivers were mailed a sleep headband and received training on setting up and using the device. At-home sleep EEG was recorded for 3 consecutive nights. Participants with sensory sensitivities or those needing additional adaptation time first received a practice “mock” headband (ie, an identical headband with the electronics deactivated) and completed a modular desensitization protocol before sleeping with the functional headband. While this study was primarily designed for remote participation, local participants were given the option of attending an in-person study visit. The study timeline is presented in Figure 1.

**Figure 1.**
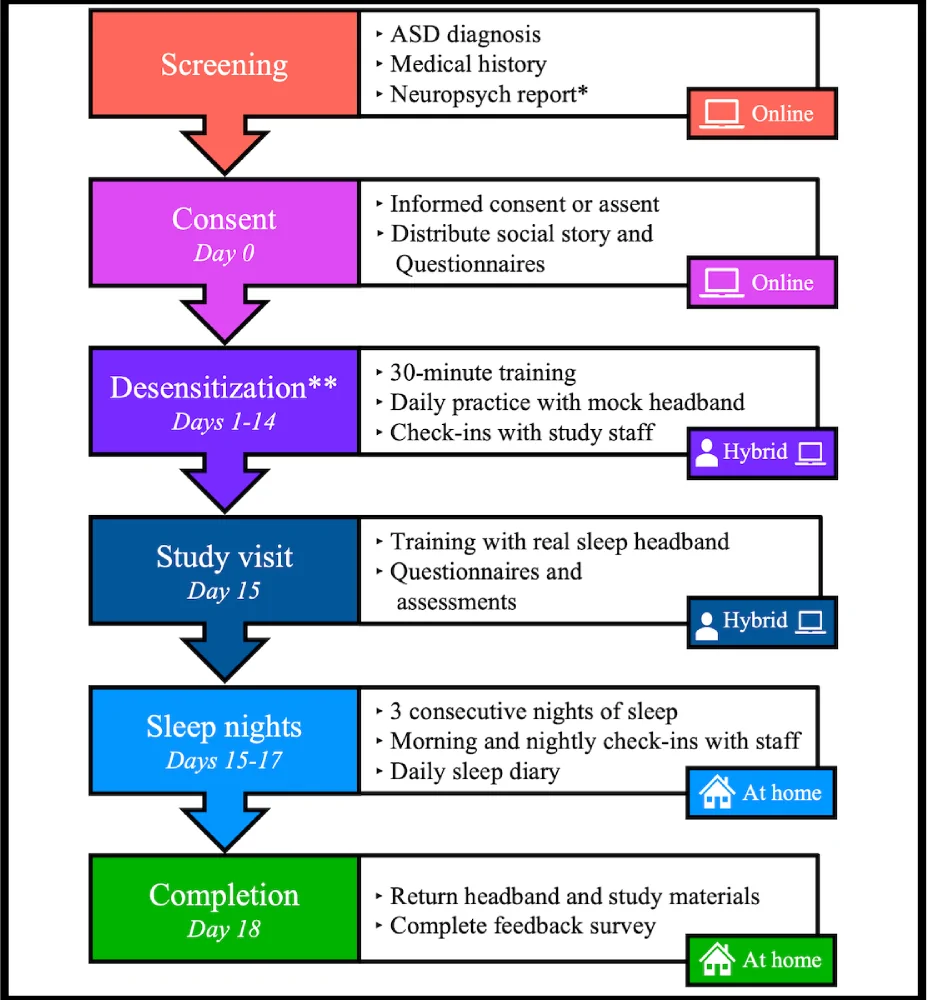
Study timeline. Interested participants/families completed online screening to confirm eligibility and provided informed consent via video call. Families who opted to start with the desensitization protocol were sent a mock headband, completed a 30-minute training call, and checked in with the team for up to 2 weeks. Once acclimated to sleeping with the headband, participants/families completed a 1.5-hour study visit either in person or remotely (hybrid), during which they completed standardized assessments and received training on using the real electroencephalography (EEG) headband. Participants slept wearing the headband for 3 consecutive nights. At the end of the study, the equipment was returned by mail in a prepaid box, and families completed a feedback survey. *Neuropsychological reports were only obtained from participants with autism spectrum disorder (ASD) or profound autism. **Desensitization was optional and intended for those who needed time to acclimate to sleeping while wearing the headband.

#### Power

Based on a study by Maski et al [27], with 72 participants per group and α=.05, we will have >90% power to detect group differences in spindle density based on estimated effect sizes (*d*=0.81). Mylonas et al [10] reported a significant negative relationship between WASO and spindle density in ASD (n=19; *r*=–0.67). Based on these findings, we have >99% power to detect correlations between spindles and sleep quality within each group.

#### Participants

In anticipation of a 10% attrition rate in participants with ASD and TD participants due to not tolerating the sleep headband and/or incomplete or poor-quality sleep data, we aimed to enroll 80 adolescents with ASD and 80 demographically matched TD peers to obtain complete data from 72 participants per group. We expected a higher, 33% attrition rate in participants with profound autism and aimed to enroll 15 to obtain complete data in 10 as a pilot sample.

#### Recruitment

Participants with ASD were recruited from the Lurie Center for Autism in Lexington, Massachusetts, and the Simons Foundation Powering Autism Research (SPARK) initiative via Research Match. Participants with ASD and TD peers were also recruited from the local community and Mass General Brigham’s online research database platform Rally. With the exception of profoundly affected autistic individuals, participants and parents/guardians of minors under 18 were required to be able to converse and respond to questionnaires in English.

#### ASD

Clinical diagnoses of ASD were confirmed through chart review, parental responses to the Social Communication Questionnaire (SCQ) [28], and the completion of the *DSM-5* (*Diagnostic and Statistical Manual of Mental Disorders* [Fifth Edition]) checklist. All participants with ASD met criteria for ASD on the *DSM-5* based on chart review or had an SCQ score of 15 or above. Participants with known genetic syndromes associated with ASD, diagnosed sleep disorders (except insomnia), poorly controlled seizures (1 or more within the last week), other neurological conditions, or poorly controlled chronic medical conditions were excluded.

#### Profound Autism

In addition to meeting criteria for ASD, participants diagnosed as having profound autism were required to also meet at least 1 of the following criteria: minimally verbal (lower than first percentile on the Expressive Vocabulary Test, Third Edition [EVT-3] [29]; or uses no words, only single words, or short phrases [2‐3 words] on a daily basis per parental report), intellectual disability (nonverbal intelligence quotient [NVIQ]<70), or significant maladaptive behaviors (total score on the Aberrant Behavior Checklist [ABC] [30]>49).

#### TD

TD participants were eligible if they did not meet *DSM-5* criteria for ASD and had none of the following: diagnosed neurodevelopmental disorder (eg, attention-deficit/hyperactivity disorder [ADHD]); a first-degree biological relative with ASD; an SCQ score of 15 or above, or a diagnosed psychiatric disorder (eg, currently medicated for depression or anxiety); sleep disorder (except insomnia), neurological disorder (including seizures); or a poorly controlled chronic medical condition expected to affect sleep.

#### Screening

Interested families completed a screening call with the study team to determine eligibility. Parents (or participants 18 or 19 years old who were not profoundly affected) were asked about their child’s medical history, including any diagnoses (eg, ASD, genetic syndromes, anxiety, depression, and epilepsy), medications, and sleep. Participants were also screened for diagnosed sleep disorders, such as sleep apnea, periodic limb movement disorder, or other parasomnias. Participants were screened for undiagnosed sleep apnea using either the Pediatric Sleep Questionnaire (PSQ; ages 12‐17 years [31]) or the STOP-BANG [32] (ages 18-19 years). Parents completed the SCQ [28] and *DSM-5* checklist to confirm their child’s ASD diagnosis or lack thereof (for TD). Independent adults (ages 18-19 years) without parental involvement were administered the Autism-Spectrum Quotient ([33]) and *DSM-5* checklist to confirm ASD diagnosis.

Gathering additional information about participants prior to participation can help researchers anticipate potential barriers and increase the likelihood of successful study completion [34,35]. If desensitization was needed, parents completed a more in-depth screening interview about their child’s expressive language skills, maladaptive behaviors that may occur during visits (eg, elopement or self-injury) or while practicing with the headband, sensory sensitivities (eg, difficulty tolerating things on their head) or sensory-seeking behavior (eg, mouthing), and motivators that might help their child succeed (Multimedia Appendix 1) [34,36,37]. This information helped study staff personalize the desensitization training and prepare for in-person visits. For example, familiar strategies, such as timers, visual schedules, and sticker charts, could be leveraged to encourage practice with the mock headband, and preferred rewards could be offered during breaks, after completing each desensitization module, and after completing the study. In-depth screening can also help researchers predict obstacles or challenges that individuals might encounter during the study and prepare mitigation strategies (eg, providing a reward for not mouthing the device or an alternate object that can be mouthed instead [34,36,37].

### Ethical Considerations

This study was approved by the Mass General Brigham Institutional Review Board (protocol 2021P002832; 2023P000239). The research was conducted in accordance with the ethical principles outlined in the Declaration of Helsinki. Informed consent and assent were obtained at study onset by the participant (18-19 years old) or a parent/guardian (<18 years old). Written assent was also required for participants under 18 years. In the profound autism group, a parent or legal guardian provided informed consent regardless of their child’s age, and participants provided assent when possible.

Participant data were deidentified prior to analysis and stored on secure, password-protected institutional servers accessible only by authorized study personnel. Participation was voluntary, and participants or their parent/guardian could withdraw from the study at any time without penalty. Participants received monetary compensation for their time.

### Headband Selection

We considered several commercially available EEG headbands, some of which have shown good agreement with PSG for sleep staging [25]. Our evaluation criteria included price, electrode montage, availability of raw data, accuracy of characterizing sleep architecture and sleep physiology, comfort, ease of setup and use, and automatic remote data upload. We also considered durability and the presence of detachable components given the possibility of participants mouthing, throwing, or banging the headband. We compared 3 headbands against PSG for sleep staging and spindle and SO detection. Across devices, sleep staging agreement with PSG was comparable to reported interscorer reliability [38]. Dreem 3 (DREEM Inc, acquired by Beacon Biosignals Inc in 2023) showed good agreement for spindle and SO detection.

Dreem 3 has 3 frontal (FP1, F7, F8) and 2 occipital electrodes (O1, O2), along with an accelerometer. Data are stored locally on the headband and automatically uploaded to the company’s server when the headband is charged. Dreem 3 includes proprietary algorithms for assessing signal quality, sleep staging, and estimating body position and the proportion of time the headband was not worn. Researchers can access data through the Dreem 3 web-based platform. Since model redesigns and new devices continually change the landscape of commercially available EEG headbands, researchers should consider which headband best fits their needs.

### Desensitization

Participants were offered an optional 2-week desensitization protocol to acclimate to the headband. Families practiced five modules at home with the mock headband: (1) the participant touches the headband, (2) a family member models wearing the headband, (3) the participant wears the headband for at least 5 minutes, (4) the participant lays down wearing the headband for at least 5 minutes, and (5) the participant wears the headband overnight while sleeping (Figure 2). At the beginning of desensitization, families attended a 30-minute meeting (either in person or virtual) with the study team and a behavioral specialist. They received a social story outlining the 5 modules (Multimedia Appendix 2) and were introduced to the headband. The study team observed the participant’s initial response to the headband and had them attempt the first 4 modules to determine which they should begin with. Families were given instructions and tips-and-tricks for practicing at home (S1 in Multimedia Appendix 3). Families were encouraged to practice at least 3 times a day and were asked to log their child’s progress, including which module they practiced, practice duration, and their child’s level of comfort and cooperation (S2 in Multimedia Appendix 3). To avoid developing a negative association to the headband, families were asked to stop practicing if their child became uncomfortable or resistant and allowed the study team to help troubleshoot. Since cooperation and comfort may look different for each child, families completed a worksheet identifying behaviors they observe when their child is uncomfortable/resistant in other situations to inform their rating of comfort/cooperation (S3 in Multimedia Appendix 3). Once the child successfully completed a module 3 times in a row (modules 1‐4), they advanced to the next module. Desensitization was complete once the child slept while wearing the headband overnight 1 time (module 5).

The study team monitored progress through daily text check-ins (Multimedia Appendix 3) and a phone check-in after the first week. Based on the child’s initial tolerance of the headband and the family’s available practice time, desensitization could be extended up to a month. Children with greater sensory sensitivities may need more time to adapt to new equipment [35,36,39]. Some may also benefit from breaking down the desensitization modules into smaller intermediate steps. Providing opportunities for breaks and moving at the pace of the child may further improve data quality [34]. While a flexible desensitization period helps support participants, parents may have demanding schedules and limited time for the study. The study team sought to balance regular participant engagement to provide support and encourage participation with not overburdening families with excessive contact. Check-ins were adapted to their schedule, preferred method, and frequency.

**Figure 2.**
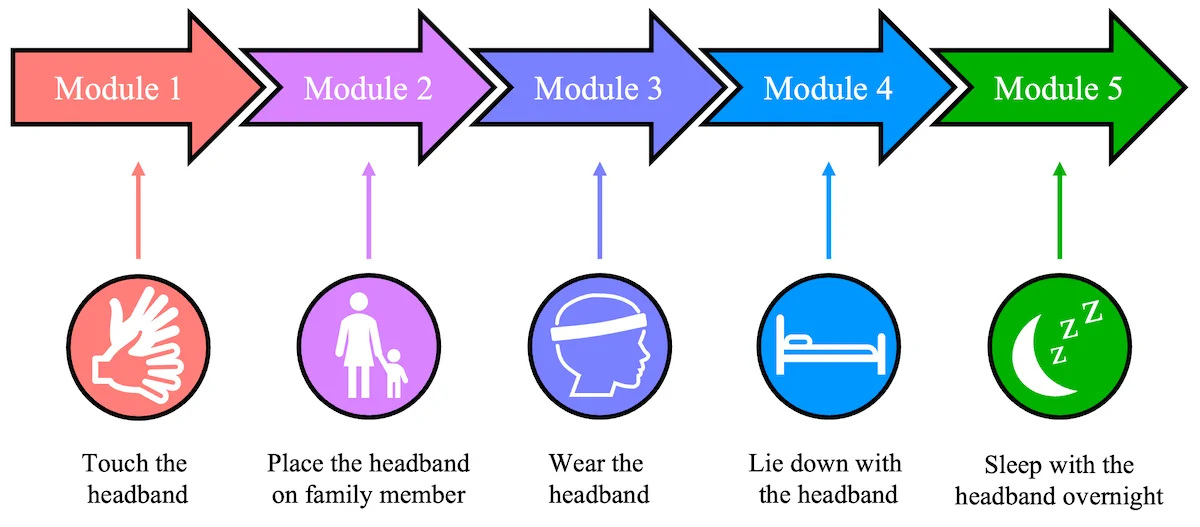
Desensitization modules.

### Study Visit and Headband Training

#### In-Person Visit

Participants with ASD from the local area were given the option to complete an in-person study visit. All participants with profound autism came in person. To prepare for the visit and upcoming sleep recordings, families were emailed an individualized social story (Multimedia Appendix 2). During the visit, participants were trained to use the EEG headband, including downloading the phone app, placing the headband correctly, checking the signal quality, starting/stopping the recording, and recharging the headband. The study team inspected the headband fit to ensure that it was placed correctly and fit snugly. They provided extenders if it was too small and gave pointers to promote good quality data (eg, removing hair from underneath the headband or wearing a cloth headband over the EEG headband to keep it in place). Printed set-up instructions and nightly/morning checklists were provided for reference (Multimedia Appendix 4). Participants and their families also completed questionnaires and neuropsychological assessments to assess their sleep [40-42], sensory sensitivities [43-45], language skills [30], and maladaptive behaviors [30] (Table 1). The Stanford-Binet Intelligence Scales [46] or Leiter International Performance Scale, Third Edition [47] was administered to measure full-scale (verbal and nonverbal) or nonverbal IQ.

**Table 1.** Questionnaires and assessments.

Domain	Autism spectrum disorder	Profound autism
Sleep	Children’s Sleep Habits Questionnaire (CSHQ)^a^ [42] Pittsburgh Sleep Quality Index (PSQI) [40] PROMIS^b^ Sleep Disturbance [41] PROMIS Sleep-Related Impairment [41]	Children’s Sleep Habits Questionnaire (CSHQ)^a^ [42]
IQ	Stanford-Binet Intelligence Scales, Fifth Edition (SB-5) [46]	Leiter International Performance Scale, Third Edition [47]
Language	N/A^c^	Expressive Vocabulary Test, Third Edition (EVT-3) [29]
Sensory	Sensory Profile Questionnaire^a^ [43] Glasgow Sensory Questionnaire [44] Adolescent/Adult Sensory Profile (AASP) [45]	Sensory Profile Questionnaire^a^ [43]
Maladaptive behaviors	N/A	Aberrant Behavior Checklist (ABC)^a^ [30]

a Completed by participant’s parent.

b PROMIS: Patient-Reported Outcomes Information System.

c Not applicable.

#### Remote Training

Remote participants were mailed the EEG headband and printed instructions, checklists, and a sleep diary. Study staff conducted a headband training session via video call. When possible, nonverbal IQ estimates of participants with ASD were obtained from a previous neuropsychological report or an individual education plan.

### Overnight EEG Acquisition and Sleep Diary

Participants slept with the headband for 3 consecutive nights at home. The first night was considered an adaptation night. We aimed for weeknights to have a more consistent sleep schedule. Each evening, study staff offered technical support for headband setup and placement by video call, phone call, or text message. Each morning, participants and/or their families stopped the recording, charged the headband, and completed a sleep diary and questionnaire about headband comfort and usability (Multimedia Appendix 4). When possible, participants with profound autism completed questionnaires about the comfort of the headband that were adapted by using pictures and icons (Multimedia Appendix 4). Sleep diaries were completed by their parents.

### Data Quality Evaluation

Each morning, the study team downloaded raw EEG and accelerometer data from the Dreem 3 platform to assess data quality. We checked that (1) the raw EEG and accelerometer data were an appropriate length, (2) the signal looked physiological rather than artifactual, (3) sleep diary responses were consistent with the EEG recording, and (4) the headband remained on throughout the night. (If the headband came off for more than 10 minutes, sleep quality and architecture were not calculated.) The EEG data are sleep staged using U-Sleep [48] and then preprocessed using Luna [49] to identify epochs containing artifacts. Channels with >40% epochs identified as artifactual are flagged as being poor quality and removed. The whole-night time-frequency spectrograms aligned with the participant’s hypnogram are inspected, as are the power spectra of NREM stage 2 (N2) and NREM stage 3 (N3) sleep before and after preprocessing. Usable records have at least 1 channel with good quality data, and at least 30 minutes of artifact-free N2 and N3 sleep (see Figure 3 for the data quality checklist). If the participant reported that the headband was uncomfortable or if the data were of poor quality, the study team contacted the family to troubleshoot. Any medications taken were documented in the sleep diary each night.

**Figure 3.**
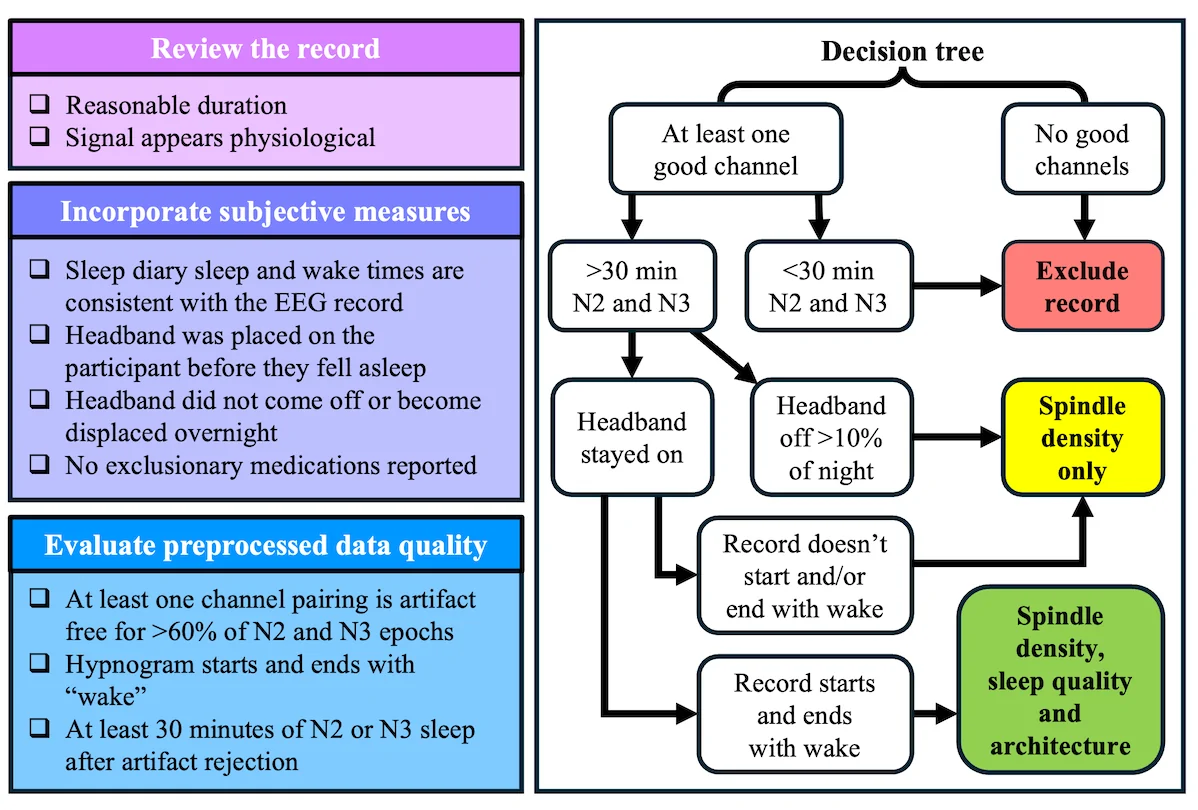
Electroencephalography (EEG) quality checklist. The study team visually evaluated EEG data quality each morning along with responses to the sleep diary and questionnaires. They then preprocessed the data to ensure it was usable. Measuring sleep quality and architecture requires both the headband to stay on the head all night and good EEG channel quality. Sleep oscillations of interest (eg, spindle density) can be reliably measured if there is at least 30 minutes of artifact-free stage 2 (N2) and stage 3 (N3) sleep. If data quality was poor, the research team contacted the family to troubleshoot. If there were no usable records after 3 nights, participants were given the option for a fourth night.

### Study Completion

At the end of the study, participants returned all equipment by mail in a provided prepaid box along with a return label, packing tape, and packing list. In addition to monetary compensation, participants received a personalized completion certificate that includes a sleep spindle recorded during the study (Multimedia Appendix 5) and a copy of “Why Sleep,” an article about the functions of sleep written for children [50]. Families also had an opportunity to provide feedback about their study experience.

### Statistical Analysis

The primary analyses will include all available recording nights except the adaptation night, unless it is the only night with usable EEG data. Analyses will be restricted to N2 and N3 sleep. Within each night, spindle measures will be averaged across all usable channels. The primary outcomes include spindle density (number of spindles/min), spindle amplitude, spindle-SO coupling phase, and coupling consistency. Secondary outcomes will include spindle duration and spindle frequency. Sleep spindle measures will be compared between groups using mixed-effect models with diagnosis; night; age; sex; and the interactions of diagnosis by age, sex, and night as fixed effects. Subject will be included as a random intercept to account for repeated observations within individuals. We will also use linear regression to predict objective sleep quality (WASO and sleep fragmentation index) from spindle density (averaged across nights) and group, controlling for age and sex. We will check for model adequacy and violation of the homoskedasticity assumption by looking for curvature and “fan-shaped” residual plots. Residual normality will be assessed using Q-Q plots. We will check for normality of random effects using the standard diagnostic procedures implemented in R [51].

We will also evaluate the relationships between spindle density and clinical features. We hypothesize that higher sensory sensitivities (assessed via the Adult/Adolescent Sensory Profile or Parent-Report Sensory Profile) will be associated with lower spindle density based on evidence linking both to thalamic reticular nucleus–mediated thalamocortical dysfunction [52-54]. We will repeat all analyses in the subset of participants with ASD who were not taking psychotropic medications to exclude a medication confound.

## Results

This study was funded by the Simons Foundation Autism Research Initiative (SFARI) and approved by the Mass General Brigham Institutional Review Board in November 2021. After validating the headbands against PSG, data were collected between July 2023 and November 2025. A total of 153 participants (ASD: n=80; TD: n=68; profound autism: n=5) were enrolled, and 146 participants (ASD: n=77; TD: n=65; profound autism: n=4) completed the study and produced at least 1 usable night of sleep data. Data analysis is underway, and the results will be submitted for publication in November 2026.

## Discussion

### Considerations to Enhance Success

Due to the heterogeneity of ASD, a one-size-fits-all approach to sleep studies may limit who is able to participate and restrict sample sizes. To promote inclusion and achieve larger samples, several strategies can be implemented to accommodate individuals with greater support needs. For example, involving a behavioral specialist with expertise in ASD in study design and execution can facilitate data collection and improve the participant’s experience. The specialist can recommend modifications and tailor procedures to each participant’s needs [36] and provide behavioral training to prepare study staff for successful participant interactions. Considering intellectual and communication abilities [35,36] is also necessary to determine which questionnaires and assessments are appropriate. If subjective report measures are not feasible, parental report or questionnaires with pictures may be alternatives. Neuropsychological assessments that do not rely on receptive or expressive language, such as the Leiter [47], may be better suited to characterize cognitive function. Social stories, visual schedules, and videos can clarify study procedures, reduce uncertainty, and ease transitions between tasks [34,39,55]. Study instructions should be concise, use simple language, and incorporate visuals or modeling. Providing materials in different formats—such as video and printed social stories—allows families to choose the most effective approach for their child. Consulting with families during protocol development and personalizing the research experience as much as possible [37] will promote successful participation.

Collecting sleep data in the home reduces geographical and practical constraints, thereby enhancing accessibility and inclusivity. This strategy also enables studies of rare populations. For example, we are using this protocol to study sleep in individuals with genetic syndromes associated with ASD, specifically Phelan-McDermid, Fragile X, and 16p11.2 deletion syndromes. These rare genetic syndromes account for <0.02% of the population, and families are geographically dispersed. Although sleep disturbances are highly prevalent in these syndromes [56-59], their sleep physiology has seldom, if ever, been studied. At-home sleep monitoring has allowed us to expand our recruitment across the United States. Our desensitization protocol has also facilitated greater participation.

### Limitations

Limitations to this protocol include its reliance on self-report and/or parental-report of symptoms to confirm ASD diagnosis, rather than the ADOS-2, the gold-standard assessment. We omitted the ADOS-2 because it must be administered in person and is incompatible with a remote study design. Our study also includes individuals with high support needs who may not meet full criteria for profound autism [60]. While we believe the materials presented here may still be useful for this group, researchers specifically studying profound autism may want to add a scale of adaptive functioning, such as the Vineland Adaptive Behavior Scales, Third Edition (VABS-3) [61] to their screening procedures. Finally, while this protocol improves inclusivity for many families, those without access to Wi-Fi or who have difficulty with the technology may decline participation. Continued efforts are needed to further improve accessibility.

### Future Work

The methodology described here can be adapted for a wide range of other conditions and research goals beyond ASD. For example, this approach is well suited for longitudinal studies that track sleep across typical and atypical development or characterizing sleep in other neurodevelopmental disorders. Although wearable EEG devices allow researchers to study sleep in disorders like apnea, narcolepsy, and epilepsy remotely, diagnoses still require hospital or laboratory-based PSG. In these cases, our desensitization protocol, social stories, and other practice materials could be adapted to help patients acclimate to the sensory components of the PSG equipment and the hospital environment prior to their study, increasing the likelihood of a successful sleep study. Similar protocols could also be developed for increasing the tolerability of other medical procedures such as physical exams, blood draws, vaccinations, and imaging (eg, EEG, magnetic resonance imaging [MRI], and computed tomography). Integrating desensitization methods into clinical practice could improve both patient experience and quality of care.

Our future studies will benefit from participant feedback about desensitization training, headband comfort and usability, and overall study experience. Based on feedback, we hope to streamline surveys and materials into a smartphone app, providing families with a centralized and easily accessible platform for practice logs, sleep diaries, checklists, surveys, and reminders. This will reduce burden on families and researchers and facilitate individualization to encourage participation.

### Conclusions

Large-scale sleep studies in ASD present unique challenges but are crucial for developing and evaluating interventions for poor quality sleep. Here, we describe an approach to studying sleep oscillations in individuals with ASD using a commercially available EEG headband worn overnight at home. The primary aim of the study is to characterize sleep quality, architecture, and physiology in a large sample of adolescents with autism, including those who are profoundly affected. As the sleep oscillations that we measure map onto specific neural circuits, this study will elucidate the neural circuitry underlying abnormal sleep physiology in ASD and test the hypothesis that it contributes to poor sleep quality. This protocol enables larger, more inclusive samples in research and clinical trials aimed at developing and evaluating mechanistically targeted treatments for sleep disturbances in ASD.

## Supplementary material

10.2196/105941 Multimedia Appendix 1 Phone screen for participants with profound autism.

10.2196/105941 Multimedia Appendix 2 Social stories.

10.2196/105941 Multimedia Appendix 3 Desensitization materials.

10.2196/105941 Multimedia Appendix 4 Checklists and sleep diaries.

10.2196/105941 Multimedia Appendix 5 Completion certificate.

10.2196/105941 Peer Review Report 1 Peer review report.

## Abbreviations

ABC: Aberrant Behavior Checklist
ADHD: attention-deficit/hyperactivity disorder
ASD: autism spectrum disorder
*DSM-5*: *Diagnostic and Statistical Manual of Mental Disorders* (Fifth Edition)
EEG: electroencephalography
EVT-3: Expressive Vocabulary Test, Third Edition
MRI: magnetic resonance imaging
NREM: nonrapid eye movement sleep
NVIQ: nonverbal intelligence quotient
PSG: polysomnography
PSQ: Pediatric Sleep Questionnaire
SCQ: Social Communication Questionnaire
SFARI: Simons Foundation Autism Research Initiative
SO: slow oscillation
SPARK: Simons Foundation Powering Autism Research
TD: typically developing
VABS-3: Vineland Adaptive Behavior Scales, Third Edition
WASO: wake after sleep onset
