# Supplementary material for: Measuring Sleep at Home in Adolescents With Autism Using EEG Headbands: Protocol for a Remote Study
Source: JMIR Res Protoc. 2026 Oct 30;15:e105941. doi: 10.2196/105941 (PMC13721737; doi:10.2196/105941)
Supplement: Supplementary file 1 [file resprot-v15-e105941-s006.pdf]

Reviewer #1

This is an important proposal that intends to measure sleep at home using commercially available PSG-like systems that have the ability to measure scalp electric potentials. The main focus is on extracting measures that characterize sleep spindles (e.g., their number, timing, and concordance with slow oscillations) and comparing them between 12-18 yo adolescents with ASD versus controls. The authors suggest that ASD may be caused by abnormal function or development of the Thalamic Reticular Nucleus (TRN) and that these abnormalities may explain not only sleep problems in ASD, but also a wide variety of other symptoms including social communication difficulties. I found this part of the proposal to be extremely speculative with very little supporting evidence. The authors note a couple of fMRI studies, which do not seem to have the ability to provide relevant data for this specific mechanism. Furthermore, the presented preliminary data shows a considerable overlap in spindle characteristics across ASD and control groups (very small sample). While there may be some significant differences in between-group comparisons, the authors do not discuss the large heterogeneity across subjects, which is evident in participants of both groups. I don't think there is any existing evidence to suggest that this heterogeneity will be associated with cognitive abilities or ASD symptoms. Indeed, findings from other studies have reported mixed results regarding spindle abnormalities in ASD. To further study the potential role of sleep spindles in ASD they propose to validate PSG-like commercial systems for measuring sleep at home and then collect such data from 90 ASD and 90 control participants 12-18yo. This is definitely a worthwhile endeavor that has the potential to inform not only about spindle abnormalities, but also regarding general sleep architecture and other potential sleep problems of adolescents with ASD. Validation of the systems with only 5 NT participants seems a bit concerning as there is so much between-subject variability. There was also not enough detail regarding how validation would be performed. It was not clear what exactly is the output of each PSG-like system (temporal resolution? number of channels?) and how each system would be compared with their lab-based PSG system. There were also very limited details regarding measures of sleep other than spindles. Unclear if validation will be performed with NR participants who are of the same age as those tested in Aim 2. Feasibility of use at home will then be assessed with only 10 ASD participants who seem to be randomly selected. This is also a point of concern. There does not seem to be a protocol for identifying participants who are more likely to complete the sleep assessments and tolerate the PSG-like recordings. For example, it may be good to quantify existing sleep disturbances and sensory sensitivities with questionnaires as part of the feasibility assessment. What will the investigators do if tolerance is low and participants take off the systems in the middle of the night? Such options are not considered with enough depth. The second aim of the study is to collect data from 90 ASD participants who will be recruited through SPARK. They will then be assessed remotely (packages sent through the mail) while using video conferencing to interact with the families and troubleshoot potential problems. I think this is a considerable weakness of the proposed study. There are many things that may go wrong in setting up these systems and ensuring that they are collecting data properly on each night of the study. I would suggest that it would be important to first show feasibility with 30-40 local participants in order to determine whether it is reasonable to run a study like this remotely. While the topic of research is very exciting and the goals are timely and highly warranted, the planned execution raises several major concerns regarding feasibility. I think this project would be a good candidate for "pilot study" funding rather than a full 3-year research program of this magnitude.

Reviewer #2

The proposed project will evaluate sleep oscillations in ASD with the preliminary goal of identifying the best wearable device for recording these oscillations at home (Aim 1) and then using this formation to characterize abnormal sleep physiology in ASD (Aim 2). The proposal has several

strengths including focus on a key neurological function that is often impaired in ASD, has links to molecular pathology in ASD, and causes significant functional impairment. The evaluation of sleep spindles, which have been linked to neurobehavioral function and may be a useful treatment target in future studies, is a strength. The preliminary data support the application by showing that wearable devices can be used to score sleep oscillations, demonstrating feasibility of data collection from the ASD adolescent population, providing evidence of abnormal sleep coordination in non-REM sleep in ASD- although the results for spindles and memory appeared to be a bit counter intuitive in ASD. Power analyses seem reasonable for both aims. The research team has significant experience with conducting sleep studies in ASD, which is not a trivial endeavor. Weaknesses include: a) it would have been useful to see more data on feasibility and whether the devices impair sleep in ASD. In prior sleep studies, it has often been the case that longer lead-in periods and longer observation periods are needed to obtain reliable sleep data. Justification for the very short period (3 nights for two consecutive weeks) would be useful but for a feasibility study may be a reasonable place to start. Unfortunately, though, without attempting more observations (more nights or more weeks) it may not be possible to fully evaluate the best approaches to getting highly reliable measurements for characterizing individual differences and tracking change in future treatment trials. b) the use of 80 ASD and 90 NT seems a bit off to this reviewer. The reason is that ASD is likely to produce greater heterogeneity (of interest) than the NT group. It would seem to make more sense to have a smaller NT group and a larger ASD group to better characterize heterogeneity and individual differences in the ASD group. c) Sensory profile may be more useful than the Glasgow sensory questionnaire in ASD samples. d) more info on how sleep oscillations (spindles) might relate to particular aspects of ASD would be useful as this would provide more information to use these measurements in future clinical trials. e) statistical analysis plan needs more info about how missing data will be handled. Listwise deletion is not useful in these types of studies and the specific methods for handling missing nights/data points needs to be clearer. f) removing the ASD plus ID participants may be reasonable in this case but it does reduce generalizability of the study and is unfortunate because these folks are most eager for clinical trials and having objective measures is most needed in this group. This group also has more sleep difficulties. 2. Responsiveness to the Human Cognitive and Behavioral Science RFA: Does the work lay critical groundwork for future translational efforts? If successful this project could produce a wearable solution that could score sleep oscillations which relate to neurobehavioral function and could be useful in future clinical trials.

Reviewer #3

This proposal aims to validate the use of commercially-available wearable EEG sensors and associated biomarkers based on sleep spindles identify abnormal sleep oscillations in ASD to illuminate pathophysiology and guide treatment development. From a technology perspective the proposal is very comprehensive and outlines an appropriate validation methodology for the technology with preliminary results reported suggesting a good chance of success. I am less familiar with the literature relating to using sleep for the assessment of ASD pathophysiology so I am not confident to evaluate its potential impact.

Response to review

We thank the reviewers for their thoughtful and helpful critiques, which we have outlined below in italics, followed by our response and a description of any changes to the protocol. We acknowledge and appreciate their very reasonable concerns about feasibility and hope we have addressed them satisfactorily.

Reviewer #1

The authors suggest that ASD may be caused by abnormal function or development of the Thalamic Reticular Nucleus (TRN) and that these abnormalities may explain not only sleep problems in ASD, but also a wide variety of other symptoms including social communication difficulties. I found this part of the proposal to be extremely speculative with very little supporting evidence. The authors note a couple of fMRI studies, which do not seem to have the ability to provide relevant data for this specific mechanism. Furthermore, the presented preliminary data shows a considerable overlap in spindle characteristics across ASD and control groups (very small sample). While there may be some significant differences in between-group comparisons, the authors do not discuss the large heterogeneity across subjects, which is evident in participants of both groups. I don't think there is any existing evidence to suggest that this heterogeneity will be associated with cognitive abilities or ASD symptoms.

We agree that the links between TRN dysfunction and symptoms are more theoretical than empirical. We also agree that TRN dysfunction is unlikely to account for all the manifestations of ASD. However, as reviewed in the proposal, rodent studies provide compelling empirical evidence of links between risk genes for ASD, TRN dysfunction, sleep spindle deficits, and difficulty gating the relay of distracting sensory information from the thalamus to cortex resulting in attention deficits (Wells et al., 2016). Because of its size (at its widest, 3 mm) and location deep in the brain, it is very difficult to study the TRN and its function in humans using neuroimaging or non-invasive electrophysiological techniques. Findings in ASD of abnormal thalamocortical connectivity as measured by functional connectivity MRI, spindle deficits, and our preliminary data showing reduced coupling between thalamic spindles and cortical slow oscillations (SOs) provide further, albeit indirect, evidence of TRN-mediated thalamocortical dysfunction.

The literature examining sleep oscillations in ASD is inconsistent, likely reflecting the small sample sizes, sampling different ages, and the heterogeneity of ASD. In our preliminary data, the large variability of sleep parameters in both the NT and ASD groups likely reflects the range of ages (9-17). For example, while SO amplitude decreases across adolescence, spindle amplitude increases, and the amplitudes of both oscillations stabilize by young adulthood. The group comparisons in our preliminary data account for the range of ages. We also observed some intriguing, hypothesis-generating group differences in the relations of age to sleep oscillation parameters, suggesting that in individuals with ASD spindles and their coordination with SOs, which index the maturation of thalamocortical circuitry, develop differently. But much larger and possibly longitudinal studies are necessary to identify and understand differences in the development of sleep oscillations in ASD.

The goal of the present proposal is to enable such large home-based studies of sleep that could overcome hurdles that limit our understanding and treatment of abnormal sleep in ASD. Larger home-based studies could more definitively identify sleep abnormalities, decipher their genetic bases, understand the bases of their heterogeneity, establish their relations to clinical and cognitive manifestations, examine differences in their developmental trajectory, and facilitate clinical trials by increasing their power both by increasing sample sizes and by allowing targeted selection of participants who are most likely to benefit based on their sleep physiology.

Validation of the systems with only 5 NT participants seems a bit concerning as there is so much between-subject variability.

Because validation will be within-subjects, between-subject variability is less of a concern. Each participant will have three measurements, one with each device, and will serve as their own control. Measurements of sleep oscillations of interest to the present study (spindles and SOs)

show high variability between individuals but are highly reliable within individuals across nights, so much so that they have been referred to as 'EEG fingerprints' (De Gennaro et al., 2005; Purcell et al., 2017; Cox et al., 2018; Mylonas et al., 2020). Therefore a within-subject design allows us to attribute differences in measurements across devices to the device itself rather than to the participant.

There was also not enough detail regarding how validation would be performed. It was not clear what exactly is the output of each PSG-like system (temporal resolution? number of channels?) and how each system would be compared with their lab-based PSG system. There were also very limited details regarding measures of sleep other than spindles.

All the wearable devices have at least one frontal electrode and sufficient temporal resolution to capture spindles and SOs (DREEM: 5 channels (F7/F8/Fp2/O1/O2) at 250Hz; Hypnodyne: 3 channels (F7/F8/Fpz) at 256Hz; Philips SmartSleep: 1 channel (Fpz) at 250Hz). We will assess the reliability of each wearable device against PSG (gold standard) on *i)* sleep scoring and *ii)* oscillation detection (spindles, SOs) during N2 and N3. For sleep scoring, all available electrodes will be used and reliability will be calculated separately for each sleep stage, as is standard according to AASM guidelines (Rosenberg and Van Hout, 2013). Reliability of oscillation detection will be assessed at the PSG frontal electrode closest to the device frontal electrode based on the agreement of the detected oscillations using established methods (Warby et al., 2014). Briefly, the specificity and sensitivity of sleep scoring and oscillation detection for each device against PSG will be calculated based on the true detections, false positives and false negatives. From this we will derive an F1-score, which is the harmonic mean of specificity and sensitivity, and ranges from 0 (no agreement) to 1 (perfect agreement) with PSG.

Unclear if validation will be performed with NT participants who are of the same age as those tested in Aim 2.

Yes, validation will be performed with NT participants of the same age as those we plan to test in Aim 2. Aim 1a) Validation: We will bring in 5 NT adolescents of a range of ages spanning 12-18 yrs old since the oscillations of interest (SOs and spindles), change from adolescence to young adulthood. By including individuals at the range of ages that match our samples, we will ensure that our devices are sensitive to and accurately capture oscillations in this age range.

Feasibility of use at home will then be assessed with only 10 ASD participants who seem to be randomly selected. This is also a point of concern. There does not seem to be a protocol for identifying participants who are more likely to complete the sleep assessments and tolerate the PSG-like recordings. For example, it may be good to quantify existing sleep disturbances and sensory sensitivities with questionnaires as part of the feasibility assessment.

Thank you for these suggestions. Aim 1b) Feasibility: As part of the recruitment of participants for the feasibility study, potential participants will be screened with the Sensory Profile (as suggested by Reviewer 2, specifically the Tactile Sensitivity subscale), sleep questionnaires (Pittsburgh Sleep Quality Index; Sleep Diary), and parental interview. In practice, we will select participants most likely to tolerate wearing a device based primarily on parental and potential participant interviews, after reviewing questionnaire responses. In addition, rather than conducting the feasibility study remotely as originally planned, we will bring 10 local ASD adolescents (aged 12-18) into our lab for two sessions, one with each of the two candidate devices (selected based on findings from Aim 1a) in a counterbalanced order. At the first session, participants will be given an actigraph watch, be trained on setting up the wearable device, and then sent home for three consecutive monitored overnights. They will return to the lab for a second session, at which time

we will download actigraphy data, return the actiwatch, swap devices, interview the participant and/or their guardians about their experience with the device, and send them home for an additional three consecutive overnights, after which they will return the actigraph watch and device by mail and be interviewed by phone or video call. This will provide important information on potential issues with the device setup and tolerance and allow us to address them in person when needed. We have had success with home-based PSG studies in 9 to 17 yr olds with ASD (Maski et al., 2015).

What will the investigators do if tolerance is low and participants take off the systems in the middle of the night? Such options are not considered with enough depth

We recognize that some participants will not tolerate the wearable devices. For this reason, our power analyses incorporate an anticipated 20% attrition rate based on our home-based PSG study of this age group, which 86% of ASD and 74% of NT participants successfully completed. Whenever possible, we will make every attempt to retain participants. We will remotely monitor sleep each morning after recording and in the case of data loss, we will review procedures by phone or video call and suggest modifications for an additional night. We have successfully run both MEG/EEG afternoon nap and overnight home-based PSG studies in individuals with ASD. The wearable devices are specifically designed for comfort, ease of set-up, and repeated, long-term use at home.

The second aim of the study is to collect data from 90 ASD participants who will be recruited through SPARK. They will then be assessed remotely (packages sent through the mail) while using video conferencing to interact with the families and troubleshoot potential problems. I think this is a considerable weakness of the proposed study. There are many things that may go wrong in setting up these systems and ensuring that they are collecting data properly on each night of the study. I would suggest that it would be important to first show feasibility with 30-40 local participants in order to determine whether it is reasonable to run a study like this remotely.

In terms of costs, subject availability during the school year, and other logistics, it is not feasible to do this with 30-40 local participants. The ultimate goal is to conduct these studies in the home setting. We recognize that many things may go wrong, have a protocol for lost data (see response above), and will try to minimize this based on our in-person experiences with participants in Aim 1b. Based on our experience in Aims 1a and 1b, we will also work with device companies to optimize comfort and measurements. In Aim 1b) Feasibility, we might discover that all devices produce worse sleep quality based on actigraphy data. This may be unavoidable, but wearing a device at home will likely lead to better sleep quality than a laboratory-based study. Regardless, worse sleep quality, unless substantial, should not compromise the validity of our oscillation measurements, which are independent of sleep quality.

Reviewer #2

Weaknesses include: a) it would have been useful to see more data on feasibility and whether the devices impair sleep in ASD. In prior sleep studies, it has often been the case that longer lead-in periods and longer observation periods are needed to obtain reliable sleep data. Justification for the very short period (3 nights for two consecutive weeks) would be useful but for a feasibility study may be a reasonable place to start. Unfortunately, though, without attempting more observations (more nights or more weeks) it may not be possible to fully evaluate the best

approaches to getting highly reliable measurements for characterizing individual differences and tracking change in future treatment trials.

Please see our responses to this concern above. We agree, but also note that in addition to our overall 80% success rate in our one-night home-based PSG study in ASD and NT children and adolescents (Maski et al., 2015), we have successfully completed data acquisition in19/23 ASD and 17/18 NT young adults who wore an EEG cap and slept on their backs in an MEG scanner in three daytime nap sessions (one adaptation, one baseline, and one experimental involving a memory task before and after sleep). Importantly, we did not see any differences between sleep parameters, including sleep quality, spindles, and SOs, in either group for the adaptation and baseline visits. This is not entirely surprising since spindles and SOs are highly reliable from night to night (and nap to nap) in both healthy individuals and clinical samples (De Gennaro et al., 2005; Purcell et al., 2017; Cox et al., 2018; Mylonas et al., 2020). The devices we plan to use are designed for long-term use and should be more comfortable than either PSG or sleeping in an MEG scanner. We will be able to evaluate whether the devices themselves will produce reliable measurements from night to night based on the adaptation and two experimental nights in the first 10 ASD participants of Aim 1b. We will remotely monitor sleep each morning after recording, and if a participant had particularly poor sleep quality on the experimental vs. adaptation night, we will call to problem-solve, and request to record an additional night.

The use of 80 ASD and 90 NT seems a bit off to this reviewer. The reason is that ASD is likely to produce greater heterogeneity (of interest) than the NT group. It would seem to make more sense to have a smaller NT group and a larger ASD group to better characterize heterogeneity and individual differences in the ASD group.

We are including the first 10 ASD participants in the sample for a total of 90 per group. Based on our pilot study (Figure 2), and contrary to our expectations, during N3, despite significant group differences in mean spindle density, the groups did not differ significantly in variance (Figure 2 in the application; variance: ASD=.70, TD=1.98; p=.98). In N2, where we observed significant group differences in coupling (Figure 3b, c), the variances in coupling phase (ASD=.69, TD=.40; p=.13) and consistency (ASD=.013, TD=.008; p=.20) also did not significantly differ between groups. These were the values on which we based our power analyses, but there were also no significant group differences in variance for density or coupling in either N2 of N3. Age probably accounted for most of the within-group variability. On this basis, we will keep the sample sizes equal and recruit the same range of ages in both groups.

Sensory profile may be more useful than the Glasgow sensory questionnaire in ASD samples.

Thank you, we will substitute the Sensory Profile for the Glasgow.

More info on how sleep oscillations (spindles) might relate to particular aspects of ASD would be useful as this would provide more information to use these measurements in future clinical trials.

We plan to examine the relations between sleep oscillations and clinical measurements based on phenotyping in the SPARK Research Match sample.

Statistical analysis plan needs more info about how missing data will be handled. Listwise deletion is not useful in these types of studies and the specific methods for handling missing nights/data points needs to be clearer.

To take into account the multiple measurements for each subject and avoid listwise deletion of missing data, we will evaluate group differences in sleep quality and oscillation parameters using mixed effects models in which missing data are imputed using maximum likelihood estimation (Pinheiro and Bates, 1996).

Removing the ASD plus ID participants may be reasonable in this case but it does reduce generalizability of the study and is unfortunate because these folks are most eager for clinical trials and having objective measures is most needed in this group. This group also has more sleep difficulties.

We agree, but want to maximize our chances of success in this initial pilot study with the hope that we can apply what we learn about running home-based studies with wearable devices to inform future studies of larger and more diverse ASD samples. In addition, finding comparable controls for ASD plus ID individuals is challenging since sleep spindle density and amplitude correlate with IQ in multiple studies. This issue becomes less relevant for larger future studies focused on the genetics of sleep oscillation deficits.

Reviewer #3

This proposal aims to validate the use of commercially-available wearable EEG sensors and associated biomarkers based on sleep spindles identify abnormal sleep oscillations in ASD to illuminate pathophysiology and guide treatment development. From a technology perspective the proposal is very comprehensive and outlines an appropriate validation methodology for the technology with preliminary results reported suggesting a good chance of success. I am less familiar with the literature relating to using sleep for the assessment of ASD pathophysiology so I am not confident to evaluate its potential impact.

Thank you! We look forward to getting started.

Citations

Cox R, Mylonas DS, Manoach DS, Stickgold R (2018) Large-scale structure and individual fingerprints of locally coupled sleep oscillations. Sleep 41.

De Gennaro L, Ferrara M, Vecchio F, Curcio G, Bertini M (2005) An electroencephalographic fingerprint of human sleep. Neuroimage 26:114-122.

Maski K, Holbrook H, Manoach D, Hanson E, Kapur K, Stickgold R (2015) Sleep Dependent Memory Consolidation in Children with Autism Spectrum Disorder. Sleep 38:1955-1963.6

Mylonas D, Tocci C, Coon WG, Baran B, Kohnke EJ, Zhu L, Vangel MG, Stickgold R, Manoach DS (2020) Naps reliably estimate nocturnal sleep spindle density in health and schizophrenia. J Sleep Res 29:e12968.

Pinheiro JC, Bates DM (1996) Unconstrained parametrizations for variance-covariance matrices. Stat Comput 6:289-296.

Purcell SM, Manoach DS, Demanuele C, Cade BE, Mariani S, Cox R, Panagiotaropoulou G, Saxena R, Pan JQ, Smoller JW, Redline S, Stickgold R (2017) Characterizing sleep

spindles in 11,630 individuals from the National Sleep Research Resource. Nat Commun 8:15930.

Rosenberg RS, Van Hout S (2013) The American Academy of Sleep Medicine inter-scorer reliability program: sleep stage scoring. Journal of clinical sleep medicine : JCSM : official publication of the American Academy of Sleep Medicine 9:81-87.

Warby SC, Wendt SL, Welinder P, Munk EG, Carrillo O, Sorensen HB, Jennum P, Peppard PE, Perona P, Mignot E (2014) Sleep-spindle detection: crowdsourcing and evaluating performance of experts, non-experts and automated methods. Nature methods 11:385-392.

Wells MF, Wimmer RD, Schmitt LI, Feng G, Halassa MM (2016) Thalamic reticular impairment underlies attention deficit in Ptchd1(Y/-) mice. Nature 532:58-63.
