# Supplementary material for: Measuring Sleep at Home in Adolescents With Autism Using EEG Headbands: Protocol for a Remote Study
Source: JMIR Res Protoc. 2026 Oct 30;15:e105941. doi: 10.2196/105941 (PMC13721737; doi:10.2196/105941)
Supplement: Supplementary file 2 [file resprot-v15-e105941-s001.docx]

**Phone screen for profound autism participants.** Questions to identify challenging behaviors (e.g., mouthing, elopement) and sensory sensitivities, learn about what strategies work best for the child, and assess the child’s verbal ability.

**Profound autism phone screener**

1. How many different words does your child say on a daily basis?

- Which words do they use to communicate without prompts?
- Does your child combine words into a short phrase?

2. Does your child have an intellectual disability?
3. Does your child exhibit challenging behaviors at home?

- If yes, please indicate which behaviors your child exhibits and how frequently you see this behavior:
- What are triggers or activities that set off these behaviors?
- How long do these episodes last?
- What might help your child calm down?
- Do you think your child might exhibit challenging behaviors during the research study appointment?
  - If yes.. Would the person accompanying your child be able to manage the behaviors on their own?
  - If not, is it possible for someone else to come with you to the visit to help?

*Next, I will ask some questions about how your child might respond to the sleep headband and the different activities during the in-person visit. These questions are intended to help us learn more about your child so that we can adapt our training to best help them succeed wearing the headband.*

4. Does your child have a strong reaction to sounds, textures, or smells?
5. Does your child put objects in their mouth?

- If yes, how difficult is it to redirect this behavior?

6. Does your child have any difficulty with washing or brushing their hair?
7. Can your child tolerate things touching their head (for example, will they wear a hat, headband, headphones)?
8. Do you think your child would be able to tolerate wearing the headband for a short period of time during the day?
9. Do you foresee your child having any challenges wearing the headband overnight?
10. Do you think that your child would be able to adapt to the headband with practice and assistance from the study team?
11. What specific strategies do you think might help your child adapt to the headband?
12. What are your concerns about your child wearing the headband?
13. What are the strategies you use at home to help your child get through the day? (for example, social stories, visual schedules, timers, rewards, etc.)
14. What types of rewards or reinforcers are motivating for your child? (for example, food, music, access to technology, praise, etc.)
15. What are some of your child’s favorite activities, characters, TV shows/movies?
16. How does your child do when they go to the doctor’s office? Is this situation distressing for them? Do they do better if they know they are not getting a shot?
17. Is there anything else you would like us to know about your child before meeting them?
