# Supplementary material for: Measuring Sleep at Home in Adolescents With Autism Using EEG Headbands: Protocol for a Remote Study
Source: JMIR Res Protoc. 2026 Oct 30;15:e105941. doi: 10.2196/105941 (PMC13721737; doi:10.2196/105941)
Supplement: Supplementary file 3 [file resprot-v15-e105941-s002.docx]

**Social stories.** Social stories for desensitization training and sleep nights, tailored for S1) remote and S2) in-person participants. S3) The desensitization training social story was also adapted into a video format so that families could select what format worked best for their child.

**S1) Remote participation**

**S2) In-person participation**

**S3) Desensitization training video**

<https://drive.google.com/file/d/1JaMCK9gQm56NfnPb_w_1ty-t9diecoFw/view?usp=sharing>
