# Supplementary material for: Measuring Sleep at Home in Adolescents With Autism Using EEG Headbands: Protocol for a Remote Study
Source: JMIR Res Protoc. 2026 Oct 30;15:e105941. doi: 10.2196/105941 (PMC13721737; doi:10.2196/105941)
Supplement: Supplementary file 4 [file resprot-v15-e105941-s003.docx]

**Desensitization materials.** S1) Training Booklet given to families with the instructions for desensitization. S2) Daily practice log for parents to track their child’s progress. S3) Cooperation and Comfort Scale Individualized Examples Worksheet for parents to rate their child’s level of cooperation and comfort with the headband. S4) Text check in survey for daily check-ins with families.

**S1) Training Booklet**

**Sleep Headband Desensitization Instructions**

**Overview:**

The goal of the practice phase of the study is to sleep in the mock headband for at least 1 night. This booklet should be used as a guide to help your child reach this goal.

We are also providing a video and paper social story to help your child reach this goal. We recommend that you show your child the video and/or the social story before you start practicing and throughout the day when they are calm.

Keep in mind that there is no one path for getting through this (in terms of timeline or order of modules). Some children may have no problems wearing the headband at night, while other children might need to practice for several days. Some children may not be able to tolerate the headband at all, and that is ok.

We are here to help you and your child with the goal. Each time you practice, we will ask you to complete a journal entry using the headband practice log. We will send a text message to you each morning asking about how practice went the day before. After 1 week of practice, we will schedule a phone check-in to determine if you and your child would like to keep practicing.

We want your child to have a positive experience with the headband. If your child is showing resistance to practicing with the headband, please STOP practicing and report that at check-in the next day.

**Desensitization steps:**

1.      Touch headband

2.      Place headband on another family member and have them wear it for 5 minutes

3.      Tolerate headband on own head for 10 seconds to start, then build up to 5 minutes

4.      Lie down in bed wearing headband for 1 minute to start, then build up to 5 minutes

5.      Sleep through the night while wearing the headband

**Deciding when to start/stop:**

Each time you practice, you will rate your child’s cooperation and comfort level using the scale below.

How could you best describe your child’s cooperation and comfort level? (Scale 1 -5)

1.        Fully cooperative; Comfortable

2.        Mostly cooperative; Slightly nervous or uncomfortable

3.        Mostly cooperative; Moderately nervous or uncomfortable

4.        Less cooperative; Moderately nervous/uncomfortable, slight resistance

5.        Not cooperative; Nervous/uncomfortable/highly resistant

We recommend that you practice at least 3 times/day. You can move up a step once your child has scored a 3 or lower three times in a row.

We want this practice to be a positive experience for your child. **Any time you rate your child at a 4 or 5, let us know during your next check-in and we will reach out to you. Please stop practicing until you have a chance to speak with the team.** We might need to slow down and break the steps down further.

Once your child successfully sleeps in the headband overnight, they will be finished with desensitization.

**General TIPS for Desensitization:**

We want this to be a positive experience for your child. Introducing the headband gradually, can help your child acclimate to this device. We recommend that you show your child the video and/or the social story before you start practicing and throughout the day when they are calm.

- Stay calm, use lots of praise and encouragement, start slowly, have fun!
- Practice 3-4 times per day
- If your child is initially resistant, think creatively about how to help motivate cooperation. For example, “first headband, then iPad”. Using a visual might help your child what to expect. You also might consider allowing your child to engage in a preferred activity (e.g., iPad) while wearing the headband. This could be a good distraction.
- Use the provided tools to help prepare your child:  Watch the video with your child, show your child the visual steps.  These can be reviewed a few times/day, including right before practice sessions
- Add in other tools such as audio or visual timers or verbal prompts as needed. For example, “One more minute!” or “When the timer beeps, all done.”
- Modeling can be a very useful tool to help a child understand expectations and alleviate nervousness. If they are worried that the activity might hurt but they see another person responding calmly, they might feel less scared and more likely to try it themselves.
- If you are seeing slight resistance (rated at 4 or 5), please stop practicing and contact our clinic for next steps.

**Specific Tips for each Module:**

**Module 1: Touching the headband**

*Please remember to show your child the video and/or the social story.*

- If your child has difficulty with the first step and does not want to touch/hold the headband, we may need to break down this module further.
- Show your child that you (and other family members) are touching the headband. Facial expressions and body language should be relaxed, calm and happy during modeling.
- Explain that the headband doesn’t hurt
- Ask your child to touch the headband with one finger (or a different body part if they choose) while you hold onto it.
- Systematically increase from 1 finger to 2 fingers, etc.
- If refusing to touch the headband with their hand, see if they would be willing for it to rest on their lap.
- If your child is immediately looking to throw the headband or insert into their mouth, attempt to block and instead get an approximation of the request (e.g., a quick touch) and then try again at least a few hours later.

**Module 2: Place headband on another family member and have them wear it for 5 minutes**

*Please remember to show your child the video and/or the social story.*

- Ideally, your child will place the headband on the family member’s head and then watch as the parent wears it.
- If your child refuses to place the headband on the family member’s head, that is ok. The family member can place the headband on their own head and show the child. The goal is to smile, act nonchalant, that it is no big deal and it doesn’t hurt.

**Module 3: Tolerate headband on own head for 10 seconds to start, then build up to 5 minutes**

*Please remember to show your child the video and/or the social story.*

- Start with a family member wearing the headband. Then say, “Your turn!”
- Remind your child that this will not hurt and nothing will happen!
- Your child may want you to help put it on or may want to put it on themselves. Either is fine as long as they wear it!
- Break this down into small chunks of time as needed. The first step might only be 1-2 seconds. You can then increase by 5-10 second intervals each trial as tolerated. Once your child seems ok wearing the headband, you can increase by longer intervals (1 minute, etc.)
- Remember to use the visuals, timers, distractions and motivators!
- NOTE: If seeing resistance to wearing it even for a few seconds, let us know. We will discuss having your child practice wearing other headwear such as cloth headbands and hats.

**Module 4: Lie down in bed wearing headband for 1 minute to start, then build up to 5 minutes**

*Please remember to show your child the video and/or the social story.*

- Once your child is comfortable and able to wear the headband for up to 5 minutes while sitting up or standing, it’s time to practice wearing it lying down, ideally in their own bed. *If it’s easier for your child to start in a more neutral setting like the couch in the living room, this is ok. Ultimately, we’d like to make sure they are ok with lying down in their bed while wearing the headband.
- Family members should model this behavior first while wearing the headband. Demonstrate all the positions that your child can try: back, side, stomach, etc.
- Encourage your child to try different positions to see what they think is most comfortable. Try different pillows (in different positions) as well.
- If your child is willing to engage in any activities while lying down, e.g., watching TV, listening to music, looking at a magazine, etc. encourage these activities. You might have them start by engaging in the activity first, then add in the headband.
- Offer fidgets or other preferred items that can help to occupy their hands and distract from the headband.
- Once again, start in small increments (5-10 seconds) and increase by small increments as needed.
- Remember to use the visuals, timers, distractions and motivators!

**Module 5: Sleep through the night while wearing the headband**

*Please remember to show your child the video and/or the social story. By now your child should be comfortable wearing the headband.*

- Using the visuals, remind your child that the final step is wearing it overnight
- Go over the rules (leave it on all night, only a parent should remove)
- If helpful, family members can model going to sleep wearing the headband.
- Try to set up the pillow in the same way that helped your child tolerate the headband while lying down during practice. If that is no longer comfortable, encourage your child to try new positions
- Since this is a longer duration and the expectation is sleep, you might need to think about ways to help your child fall asleep while keeping the head band on. For example, if they are inclined to pull the headband off, maybe you think about massaging their hands with lotion (this will be relaxing & distracting and they won’t be able to remove the headband as easily!)

**S2) Headband Practice Log**

**S3) Cooperation and Comfort Scale Individualized Examples Worksheet**

**Cooperation and Comfort Scale**

Throughout the next two weeks, you and your child will be asked to practice with the headband that will be used in the study. Each time you practice, you will be asked to rate your child’s level of cooperation and comfort with the headband using the rating scale below. To help guide you with this rating, we would like you to write down specific examples of your child’s appearance (e.g., clenching teeth, increased rate of breathing, smiling) or behaviors (e.g., running away, screaming/crying, following directions) that you would consider to be consistent with each of the ratings. Each child is different, so there are no right or wrong answers.

**(1)**  **Fully cooperative; Comfortable** _______________________________________

______________________________________________________________________

**(2)**  **Mostly cooperative; Slightly nervous or uncomfortable**____________________

______________________________________________________________________

**(3)**  **Mostly cooperative; Moderately nervous or uncomfortable** ________________

______________________________________________________________________

**(4)**  **Less cooperative; Moderately nervous/uncomfortable, slight resistance**

______________________________________________________________________

**(5)**  **Not cooperative; Nervous/uncomfortable/highly resistant**__________________

______________________________________________________________________

**S4) Text check-in survey**

**Text Message Content:**

 Good morning! It is time to complete your daily check-in for the wearables sleep study.

Please complete the following survey at your earliest convenience:

[INSERT LINK TO REDCAP SURVEY]

Thank you!

**REDCap Survey Instructions/Questions/Feedback:**

 Please complete the following survey about how your child’s headband practice went *yesterday*. The survey should take <2 minutes to complete.

Please remember that the criteria to move to the next module is 3 consecutive successful trials. Successful trials are defined by a rating 1-3 on comfort and cooperation.

1. Did you practice with the headband yesterday? (Y/N)

2. What was the last module you practiced yesterday?

- Module 1 – Touching the headband
- Module 2 – Family member wearing the headband
- Module 3 – Child wearing the headband
- Module 4 – Child laying down in the headband
- Module 5 – Child sleeping in the headband

3. Please rate your child’s level of cooperation and comfort during the last practice yesterday.

- 1 = Fully Cooperative; Comfortable, not nervous
- 2 = Mostly Cooperative; Slightly nervous or uncomfortable
- 3 = Somewhat Cooperative; Moderately nervous or uncomfortable
- 4 = Less Cooperative; Moderately nervous or uncomfortable AND slightly

resistant

- 5 = Not Cooperative; Nervous or uncomfortable AND highly resistant

4. How long did they wear the headband (minutes/seconds)?

5.  How many times in a row has your child successfully completed the module?

     Remember, a trial is considered successful if your child’s comfort and cooperation

     was rated 1-3.

6. Did your child successfully sleep through the night in the headband?  (Y/N)

Possible outro messages:

Thank you for your responses!

- You have completed Module [MODULE NUMBER]! Today, please move on to practicing Module [MODULE NUMBER +1].
- Please continue practicing Module [MODULE NUMBER] today.
- Your child has completed the desensitization training. We will contact you shortly to discuss the next steps for the study.
- A member of our study team will reach out shortly to schedule a time to speak with you to get more information. Until then, **please STOP practicing with the headband**.
- Thank you for your responses! Please try and practice today.

If you have any questions, please don’t hesitate to reach out to [insert contact name] at [insert contact information].
