# Supplementary material for: Measuring Sleep at Home in Adolescents With Autism Using EEG Headbands: Protocol for a Remote Study
Source: JMIR Res Protoc. 2026 Oct 30;15:e105941. doi: 10.2196/105941 (PMC13721737; doi:10.2196/105941)
Supplement: Supplementary file 5 [file resprot-v15-e105941-s004.docx]

**Checklists and sleep diaries.** S1) Night-time/morning checklists summarizing the steps for each night and morning given to families at the beginning of the study. S2) Sleep diary and headband questionnaire about the comfort and ease of use of the headband completed each morning by participants and/or their families. S3) Sleep diary and headband questionnaire adapted for participants with profound autism. A parent completed the sleep diary and usability checklist and participants were asked to provide feedback on the headband using a picture scale whenever possible.

**S1) Night-time/morning checklists**

**S2) Sleep diary and headband questionnaire**

**Sleep Diary Morning X:**

Date:

| What time did you go to sleep last night? | How long did it take to fall asleep after lights out  (in mins)? | What time did you wake up this morning? | Compared to a regular night, how did you sleep?  (1 = much worse, 3 = the same, 5 = much better) | On a scale of 1-5, how comfortable was the headband? (1 = very uncomfortable, 5 = very comfortable) | Did you take any naps yesterday? |
| --- | --- | --- | --- | --- | --- |
|  |  |  |  |  | Yes       No  If yes, what time?    How long? |

Did you take medication last night before bed?                     YES               NO

Medication: ________________________________       Dosage:________________

Is there anything else you would like to tell us about your sleep?

**Comfort Scale Morning X:**

Please mark the number that corresponds to your experience sleeping with the device last night:

| **Rating:** | **Strongly Disagree** | **Disagree** | **Neither Agree nor Disagree** | **Agree** | **Strongly Agree** |
| --- | --- | --- | --- | --- | --- |
|  | 1 | 2 | 3 | 4 | 5 |
| I found the device comfortable to sleep with |  |  |  |  |  |
| I would be able to sleep with the device for multiple nights |  |  |  |  |  |
| I found the app easy to use |  |  |  |  |  |
| I found the device easy to take off in the morning |  |  |  |  |  |
| I found the device easy to put on before bed |  |  |  |  |  |
| The device easily connected to Wi-Fi |  |  |  |  |  |

**S3) Sleep diary and headband questionnaire (profound autism)**

**Sleep Diary**

What time did your child go to bed last night: __________:___________

How long did it take to fall asleep after lights out (in minutes): __________________

What time did your child wake up this morning: __________:___________

Compared to a regular night, how did your child sleep?

*(1 = much worse, 3 = the same, 5 = much better)*            ________________________

Did your child take any naps yesterday (please circle)?             Yes                No

If yes… What time? ______:_______    How long (in minutes)? _____________

Did your child take medication last night before bed?                  Yes               No

Medication: _____________________________       Dosage:_______________

Is there anything else you would like to tell us about your child’s sleep? _____________

______________________________________________________________________

**Comfort and Usability Scale**

*Please mark the number that corresponds to your child’s experience sleeping with the device last night, and your experience using the device. If you aren’t sure, please select N/A.*

| **Rating:** | **Strongly Disagree** | **Disagree** | **Neither Agree nor Disagree** | **Agree** | **Strongly Agree** |  |
| --- | --- | --- | --- | --- | --- | --- |
|  | **1** | **2** | **3** | **4** | **5** | **N/A** |
| My child found the device comfortable to sleep with |  |  |  |  |  |  |
| My child would be able to sleep with the device for multiple nights |  |  |  |  |  |  |
| I found the app easy to use |  |  |  |  |  |  |
| The device easily connected to Wi-Fi |  |  |  |  |  |  |
| I found the device easy to put on before bed |  |  |  |  |  |  |
| I found the device easy to take off in the morning |  |  |  |  |  |  |
