# Supplementary material for: Measuring Sleep at Home in Adolescents With Autism Using EEG Headbands: Protocol for a Remote Study
Source: JMIR Res Protoc. 2026 Oct 30;15:e105941. doi: 10.2196/105941 (PMC13721737; doi:10.2196/105941)
Supplement: Supplementary file 6 [file resprot-v15-e105941-s005.docx]

**Completion certificate.** Participants receive a completion certificate including an image of one of their sleep spindles.
